# Efficacy and Safety of Veno-Arterial Extracorporeal Membrane Oxygenation in the Treatment of High-Risk Pulmonary Embolism: A Retrospective Cohort Study

**DOI:** 10.3389/fcvm.2022.799488

**Published:** 2022-03-02

**Authors:** Hao-Yu Tsai, Yu-Tang Wang, Wei-Chieh Lee, Hsu-Ting Yen, Chien-Ming Lo, Chia-Chen Wu, Kwan-Ru Huang, Yin-Chia Chen, Jiunn-Jye Sheu, Yen-Yu Chen

**Affiliations:** ^1^Kaohsiung Chang Gung Memorial Hospital Education Department, Kaohsiung, Taiwan; ^2^Division of Thoracic and Cardiovascular Surgery, Department of Surgery, Chang Gung University College of Medicine, Kaohsiung Chang Gung Memorial Hospital, Kaohsiung, Taiwan; ^3^Division of Cardiology, Department of Internal Medicine, Chang Gung University College of Medicine, Kaohsiung Chang Gung Memorial Hospital, Kaohsiung, Taiwan

**Keywords:** pulmonary embolism, ECMO, shock, cardiac arrest, sudden death

## Abstract

**Objectives:**

Veno-arterial extracorporeal membrane oxygenation (ECMO) is increasingly used to treat high-risk pulmonary embolism (PE). However, its efficacy and safety remain uncertain. This retrospective cohort study aimed to determine whether ECMO could improve the clinical outcomes of patients with high-risk PE.

**Methods:**

Forty patients with high-risk PE, who were admitted to Kaohsiung Chang Gung Memorial Hospital between January 2012 and December 2019, were included in this study. Demographic data and clinical outcomes were compared between patients treated without ECMO (non-ECMO group) and those treated with ECMO (ECMO group). Appropriate statistical tools were used to compare variables between groups and the survival was analyzed using the Kaplan–Meier method.

**Results:**

The overall in-hospital mortality rate was 55%, in which 65% (26/40) of patients presented with cardiac arrest with a mortality rate of 77%, which was higher than that of patients without cardiac arrest (14%). There was no significant difference in major complications and in-hospital mortality between the non-ECMO and ECMO groups. However, in subgroup analysis, compared with patients treated without ECMO, earlier ECMO treatment was associated with a reduced risk of cardiac arrest (*P* = 0.023) and lower in-hospital mortality (*P* = 0.036). A log-rank test showed a significantly higher cumulative overall survival in the earlier ECMO treatment group (*P* = 0.033).

**Conclusions:**

In this retrospective cohort study, earlier ECMO treatment was associated with lower in-hospital mortality among unstable patients without cardiac arrest. Our findings suggest that ECMO can be considered as an initial treatment option for patients with high-risk PE in higher-volume hospitals.

## Introduction

Acute pulmonary embolism (PE) refers to embolic obstruction of the pulmonary artery that may contribute to cardiopulmonary failure and sudden death. High-risk PE, defined as acute PE with sustained hypotension or cardiac arrest, is associated with high mortality, ranging from 25% for patients with cardiogenic shock to 65% for those requiring cardiopulmonary resuscitation (CPR) (1). The latest 2019 European Society of Cardiology guidelines suggest immediate bolus injection of anticoagulants, systemic thrombolysis, catheter-directed therapy, and surgical embolectomy as first-line treatment in patients with high-risk PE (2). However, large numbers of high-risk PE patients can rapidly progress to cardiac arrest within one h before definitive reperfusion therapy (3). Based on the International Cooperative Pulmonary Embolism Registry, two-thirds of patients with high-risk PE did not receive thrombolysis or surgical embolectomy (4).

Veno-arterial extracorporeal membrane oxygenation (ECMO), a temporary mechanical circulatory support device, has been increasingly used over the past 20 years to treat high-risk PE. Several case series studies (5–8) have demonstrated the potential benefit of ECMO in high-risk PE patients with several indications, including haemodynamic support for profound shock or cardiac arrest, contraindication to systemic thrombolysis, failed reperfusion therapy, and cardiogenic shock after surgical embolectomy.

To date, there has been no case-control or cohort study to evaluate the efficacy and safety of ECMO in patients with high-risk PE. Therefore, the role of ECMO in the management of patients with life-threatening PE remains unclear. This retrospective cohort study was designed to (i) evaluate whether ECMO reduced in-hospital mortality in high-risk PE patients, and (ii) investigate whether earlier ECMO support improved clinical outcomes in patients without cardiac arrest. We hypothesized that the use of ECMO in patients with high-risk PE is associated with better outcomes.

## Materials and Methods

This retrospective cohort study was approved by the institutional review board of Chang Gung Medical Foundation (202000715B0). Informed consent was waived because of the retrospective nature of the study.

### Patient Selection and Data Collection

This study was performed at Kaohsiung Chang Gung Memorial Hospital, a tertiary referral teaching hospital in southern Taiwan, with an annual ECMO volume of > 100 cases. We included consecutive adult patients (aged ≥ 18 years) who were admitted to Kaohsiung Chang Gung Memorial Hospital with high-risk PE between January 2012 and December 2019. The diagnosis of PE was confirmed by the presence of thrombus on spiral computed tomography pulmonary angiography (CTPA) examination, and was indicated by (i) right ventricular (RV) overload or the presence of right heart thrombus on echocardiography, and (ii) high clinical probability of PE with confirmation by high probability ventilation/perfusion scan (V/Q scan). According to the European Society of Cardiology guidelines (2), hemodynamically unstable patients were defined as having high-risk PE, and patients with haemodynamic stability at the time of diagnosis of PE were stratified as having low-to intermediate-risk PE, and those who progressed to cardiogenic shock or cardiac arrest were also included. We excluded patients with previous RV dysfunction, chronic pulmonary hypertension, hypotension caused by acute myocardial infarction, new-onset arrhythmia, hypovolemia, or sepsis. Patients who signed a do-not-resuscitate consent were also excluded. Baseline characteristics, clinical variables, and prognostic values were collected from patients' medical records as described in our previous study (9), including age, sex, body mass index, comorbidities, symptoms, laboratory findings, evidence of RV strain on electrocardiogram (ECG), echocardiography, or CTPA. Major therapeutic strategies for the treatment of PE, including anticoagulation therapy, systemic thrombolysis, catheter-directed therapy, and surgical embolectomy, were also investigated. ECMO was performed in patients who presented with profound hypotension or cardiac arrest. ECMO placement and management have been described in detail in our previous study (9).

### Outcome Variables

The primary (efficacy) outcome was in-hospital mortality. Secondary (safety) outcomes were severe neurologic complications, severe kidney injury, major bleeding complications, and major ECMO-related complications. Severe neurologic complications were defined as severe hypoxic ischaemic encephalopathy (unconsciousness > 48 h or Glasgow Coma Scale score < 6 points after 72 h), seizure, brain hemorrhage, and brain infarction. Severe kidney injury was defined as kidney disease improving global outcomes in stage 3 (10). Major bleeding complications were reported using the International Society on Thrombosis and Haemostasis bleeding criteria (11), including (1) fatal bleeding, (2) symptomatic bleeding in a critical area or organ requiring intervention, (3) bleeding causing a 2 g dl^−1^ or more decrease in hemoglobin, or leading to the transfusion of two or more units of whole blood or red blood cells. Major ECMO-related complications were reported using the Extracorporeal Life Support Organization registry data definitions^1^, including cannulation site bleeding requiring blood transfusion or surgical intervention, and limb ischemia requiring reperfusion cannula placement, fasciotomy, or amputation.

### Statistical Analysis

Continuous variables are expressed as mean (standard deviation, SD) for normally distributed data, or as median (25–5% interquartile range [IQR]) for non-normally distributed data, and were analyzed using the Student's *t*-test or Mann-Whitney U-test, as appropriate. Categorical variables are expressed as numbers (percentages) and were compared using the chi-squared test or Fisher's exact test. Binary logistic regression was used to identify the influence of ECMO on primary and secondary outcomes, and the results are expressed as odds ratios (ORs) with 95% confidence intervals (CIs). The overall survival was analyzed using the Kaplan–Meier method, and differences in survival between groups were examined using the log-rank test. Statistical significance was set at *P* < 0.05. All analyses were conducted using the SPSS software (version 19.0; SPSS Inc., Chicago, IL, USA).

## Results

### Baseline Demographics, Clinical Characteristics, and Management Strategies

**Figure 1:**
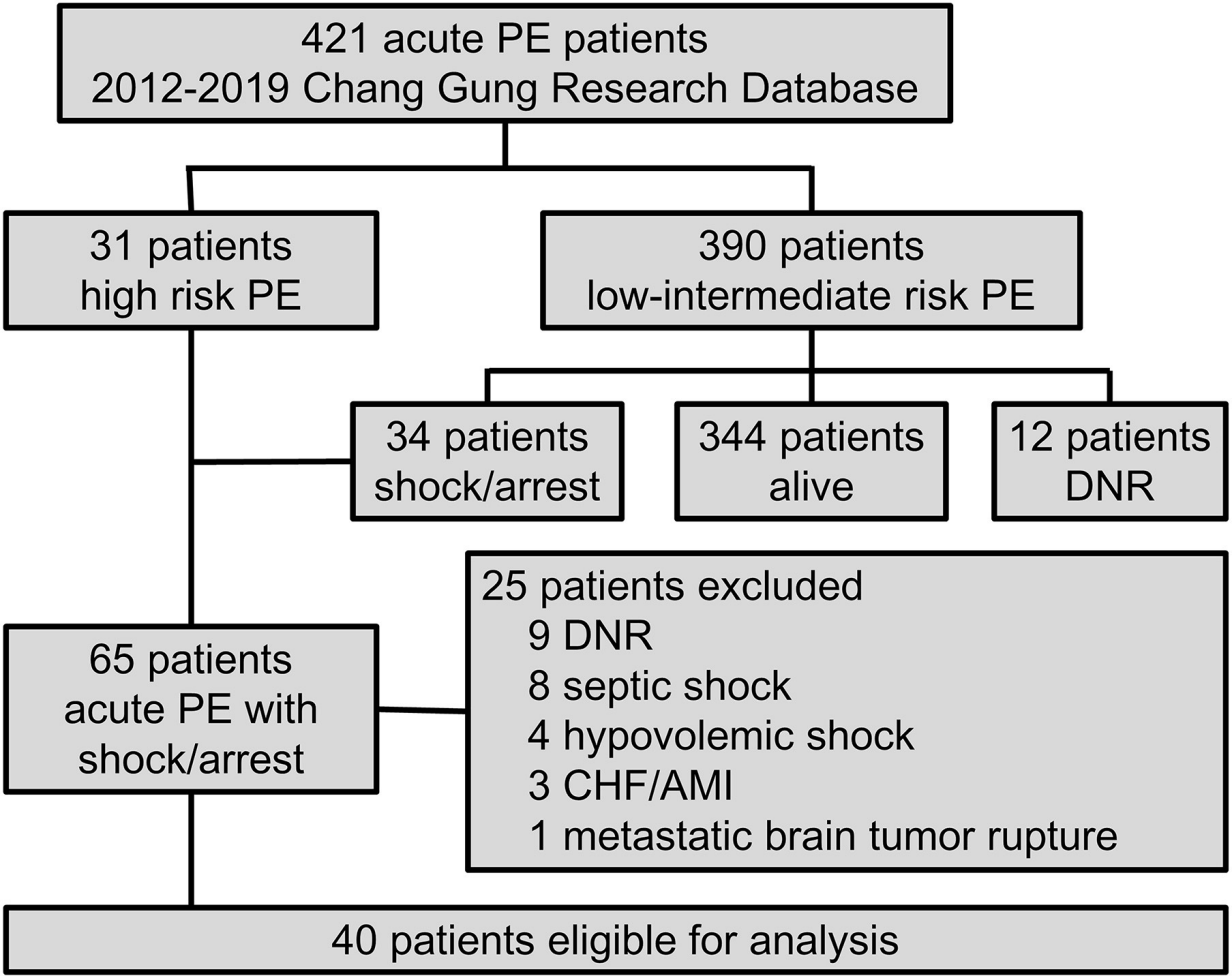
Flow chart of inclusion and exclusion criteria of this cohort study of Chang Gung Research Database from 2012 to 2019. AMI, acute myocardial infarction; CHF, congestive heart failure; DNR, do-not-resuscitate; PE, pulmonary embolism.

A total of 40 patients were included in this study (Figure 1), of whom 15 were treated without ECMO (non-ECMO group), and 25 were treated with ECMO (ECMO group). The median age was 60.5 years (IQR, 44–73 years), and 18 (45%) were female. Thirty-eight patients had a diagnosis of PE confirmed by CTPA, and two patients were diagnosed based on echocardiographic findings. Compared to the non-ECMO group, the ECMO-supported patients were more likely to be younger, male, and to have undergone major surgery within 1 month (Table 1).

**Table 1:** Baseline clinical and demographic characteristics.

	**Non-ECMO (*n* = 15)**	**ECMO** **(*n* = 25)**	***P*-value**
**Basic characteristics**			
Age (years), median (IQR)	73.0 (64–81)	52.0 (42–65)	0.002
Male gender, *n* (%)	5 (33.3)	17 (68.0)	0.033
BMI (kg/m^2^), mean (SD)	25.6 (6.0)	26.0 (4.4)	0.792
Hypertension, *n* (%)	6 (40.0)	8 (32.0)	0.608
Diabetes mellitus, *n* (%)	4 (26.7)	3 (12.0)	0.237
Hyperlipidemia, *n* (%)	3 (20.0)	2 (8.0)	0.267
Coronary artery disease, *n* (%)	3 (20.0)	4 (16.0)	0.747
Congestive heart failure, *n* (%)	2 (13.3)	2 (8.0)	0.586
Chronic kidney disease, *n* (%)	3 (20.0)	1 (4.0)	0.102
Chronic lung disease, *n* (%)	5 (33.3)	6 (24.0)	0.522
Cerebrovascular accident, *n* (%)	2 (13.3)	1 (4.0)	0.278
Liver cirrhosis, *n* (%)	0 (0.0)	1 (4.0)	0.433
**Predisposing factors for PE**			
History of VTE or recent DVT, *n* (%)	1 (6.7)	6 (24.0)	0.162
Active cancer, *n* (%)	3 (20.0)	7 (28.0)	0.572
Recent major surgery, *n* (%)	1 (6.7)	15 (60.0)	0.001
Recent major trauma, *n* (%)	0 (0.0)	3 (12.0)	0.163
Bed rest > 3 days, *n* (%)	7 (46.7)	6 (24.0)	0.138
**Initial presentation**			
Dyspnea, *n* (%)	13 (86.7)	16 (64.0)	0.120
Chest pain*, n* (%)	5 (33.3)	5 (20.0)	0.346
Cold sweating, *n* (%)	5 (33.3)	8 (32.0)	0.931
Fever, *n* (%)	2 (13.3)	6 (24.0)	0.414
Tachycardia, *n* (%)	8 (53.3)	11 (44.0)	0.567
Syncope, *n* (%)	3 (20.0)	2 (8.0)	0.267
Cardiac arrest, *n* (%)	3 (20.0)	8 (32.0)	0.411
Risk of early death (High), *n* (%)	6 (40.0)	17 (68.0)	0.083

*BMI, body mass index; DVT, deep vein thrombosis; ECMO, veno-arterial extracorporeal membrane oxygenation; IQR, interquartile range; PE, pulmonary embolism; SD, standard deviation; VTE, venous thromboembolism*.

**Figure 2:**
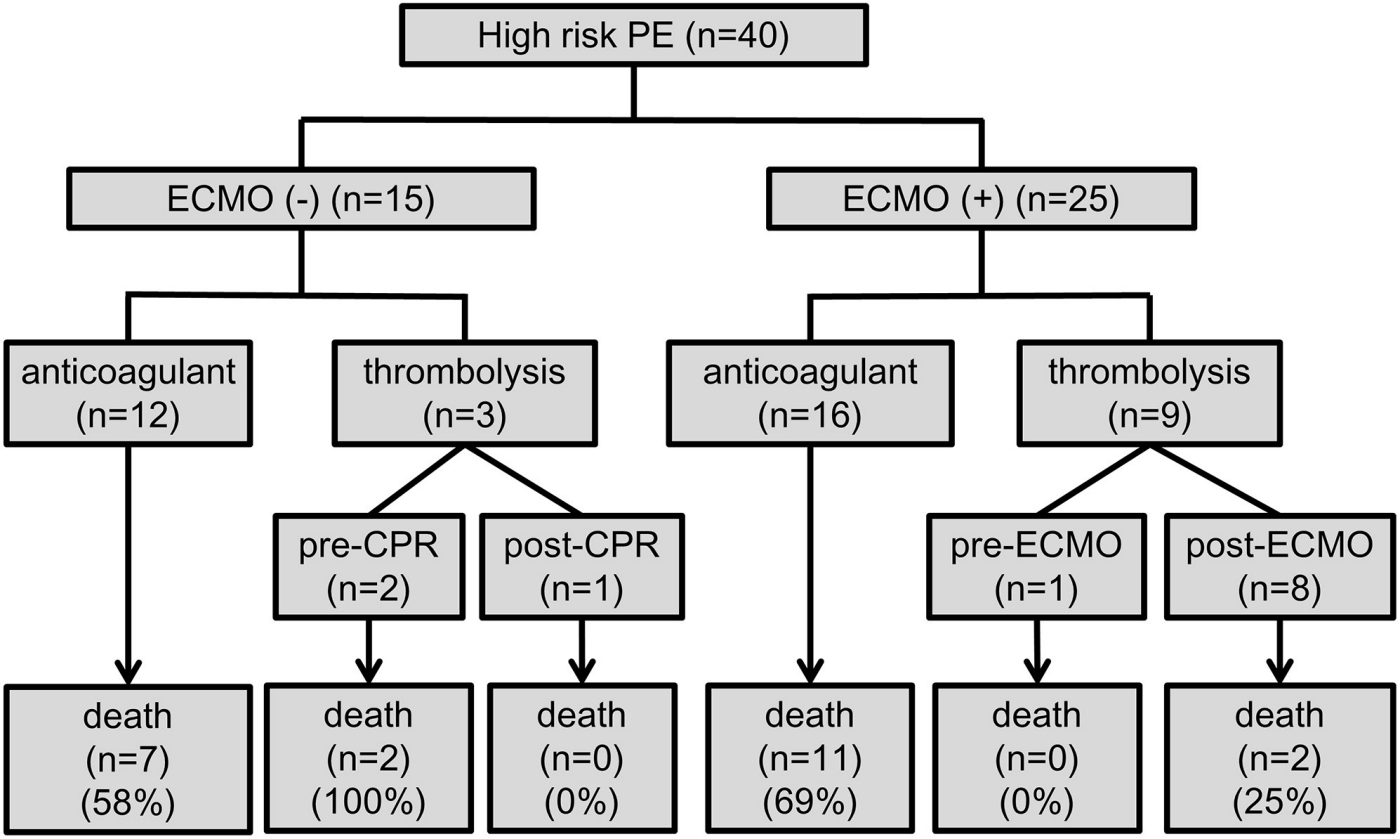
Flow chart of the treatment strategies and outcomes. CPR, cardiopulmonary resuscitation; ECMO, veno-arterial extracorporeal membrane oxygenation; PE, pulmonary embolism.

The prognostic values and management strategies are summarized in Table 2 and Figure 2. There was no difference in the incidence of cardiac arrest, RV strain, the use of ventilator and inotrope, or laboratory findings between the two groups. Three patients who received earlier ECMO treatment did not require inotropic support. ECMO was performed in 10 (25%) patients with cardiogenic shock and 15 (38%) with cardiac arrest, of whom 12 (30%) had ECMO initiated during CPR. Nineteen (48%) patients received anticoagulation therapy before haemodynamic compromise, and three (8%) patients were treated with immediate thrombolytic therapy after shock development. In total, 12 (30%) patients were treated with thrombolytic therapy in addition to anticoagulation, and 28 (70%) received anticoagulation therapy alone. Of the 28 patients who did not receive thrombolysis, 17 (43%) had at least one absolute contraindication, 6 (15%) had relative contraindications, and 4 (10%) who experienced sudden cardiac arrest (defined as an unexpected arrest within 30 min after a hypotensive episode) had no return of spontaneous circulation after 30 min of efficient resuscitation (see Supplementary Table 1). Among the patients who received thrombolysis, systemic thrombolysis was used in four patients, and catheter-directed thrombolysis was performed in eight patients (see Supplementary Table 2). None of the patients underwent a surgical embolectomy. There was no difference in the management strategy between the ECMO and non-ECMO groups.

**Table 2:** Prognostic values and management at the time of shock or cardiac arrest.

	**Non-ECMO (*n* = 15)**	**ECMO (*n* = 25)**	***P***
Cardiac arrest, *n* (%)	11 (73.3)	15 (60.0)	0.392
Sudden cardiac arrest*, *n* (%)	8 (53.3)	12 (48.0)	0.744
CPR duration (min), mean (SD)	34.8 (20.8)	44.3 (22.4)	0.281
**Right heart strain**			
RV strain on ECG, *n* (%)	6 (85.7) (*n* = 7)	16 (80.0) (*n* = 20)	0.738
RV dilation on echo, *n* (%)	2 (100.0) (*n* = 2)	19 (95.0) (*n* = 20)	0.746
RV/LV diameter ratio on CT, mean (SD)	2.5 (0.8) (*n* = 5)	1.9 (0.8) (*n* = 21)	0.100
RV/LV diameter ≥ 1.0 on CT, *n* (%)	5 (100.0) (*n* = 5)	18 (85.7) (*n* = 21)	0.369
**Laboratory finding**			
PH, mean (SD)	7.26 (0.13) (*n* = 10)	7.13 (0.24) (*n* = 22)	0.056
Bicarbonate (mmol/L), mean (SD)	17.3 (4.3) (*n* = 10)	16.8 (7.6) (*n* = 22)	0.845
SBE, median (IQR)	−5.8 (−15.7 to −4.0) (*n* = 10)	−12.5 (−19.6 to −4.1) (*n* = 22)	0.200
PaO_2_/FiO_2_ ratio (mmHg), median (IQR)	112.5 (57.9 to 262.9) (*n* = 8)	111.25 (18.45 to 185.13) (*n* = 22)	0.708
Troponin I (ng/mL), median (IQR)	0.27 (0.12 to 0.90) (*n* = 8)	0.08 (0.029 to 0.329) (*n* = 23)	0.061
Time from shock to CPR/ECMO (hours), median (IQR)	0.0 (0.0 to 2.7) (*n* = 11)	0.6 (0.0 to 1.8)	0.483
**Management**			
Mechanical ventilation, *n* (%)	13 (86.7)	25 (100.0)	0.061
Inotropic use, *n* (%)	15 (100.0)	22 (88.0)	0.163
Anticoagulation therapy	15 (100.0)	25 (100.0)	>0.999
Before shock/arrest, *n* (%)	10 (66.7)	9 (36.0)	0.060
Thrombolytic therapy, *n* (%)	3 (20.0)	9 (36.0)	0.285
Before CPR/ECMO, *n* (%)	2 (13.3)	1 (4.0)	0.278
After CPR/ECMO, *n* (%)	1 (6.7)	8 (32.0)	0.063
Pulmonary embolectomy, *n* (%)	0 (0.0)	0 (0.0)	>0.999

*CPR, cardiopulmonary resuscitation; CT, computed tomography; ECG, electrocardiogram; ECMO, veno-arterial extracorporeal membrane oxygenation; IQR, interquartile range; RV, right ventricle; RV/LV, right-to-left ventricular; SBE, standard base excess; SD, standard deviation*.

### Outcomes

The overall in-hospital mortality rate for all patients was 55% (22/40). Most deaths occurred in patients who experienced cardiac arrest, with a mortality rate of 77% (20/26), which was higher than that in patients without cardiac arrest (14%, 2/14). Univariate logistic regression analysis revealed no significant difference in major complications and in-hospital mortality (OR 0.72, 95% CI 0.20–2.64, *P* = 0.623) between the non-ECMO and ECMO groups (Table 3). Among the 25 patients with ECMO treatment, in-hospital mortality was significantly lower among patients who receive thrombolysis (22% vs. 69%, *P* = 0.025). In the non-ECMO group, the most common cause of death was unsuccessful resuscitation in six patients, followed by multiple organ failure after successful resuscitation in two patients, and profound cardiogenic shock with multiple organ failure in one patient. In the ECMO group, 12 patients died from CPR-related severe brain injury, of whom 2 had fatal brain hemorrhages and 1 experienced a fatal pulmonary hemorrhage. One severe trauma patient with lung contusion, brain hemorrhage, splenic laceration, and oliguric renal failure died during ECMO support.

**Table 3:** The primary and secondary outcomes.

	**Non-ECMO** **(*n* = 15)**	**ECMO** **(*n* = 25)**	**Unadjusted OR** **(95% CI)**	***P*-value**
**Primary outcome**				
In-hospital mortality, *n* (%)	9 (60.0)	13 (52.0)	0.72 (0.20–2.64)	0.623
**Secondary outcomes**				
Severe neurologic complications, *n* (%)	2 (22.2)^a^	13 (52.0)	3.79 (0.66–21.96)	0.137
Severe kidney injury, *n* (%)	5 (35.7)^b^	11 (44.0)	1.41 (0.37–5.45)	0.614
Major bleeding, *n* (%)	5 (45.5)	13 (52.0)	2.17 (0.57–8.19)	0.254
ECMO-related complications, *n* (%)		8 (32.0)		

*CI, confidence interval; ECMO, veno-arterial extracorporeal membrane oxygenation; OR, odds ratio*.

a *Patients who died of unsuccessful resuscitation did not take into account*.

b *Patients with end stage renal disease did not take into account*.

Among patients with severe neurologic complications, 15 (38%) patients with cardiac arrest had severe hypoxic ischaemic encephalopathy after cardiac arrest, of whom 2 had seizures and 2 had brain hemorrhages. Active bleeding requiring transarterial embolization was documented in two patients who did not receive thrombolytic therapy, and gastrointestinal bleeding requiring transfusion occurred in six patients. Among patients receiving ECMO, major ECMO-related complications occurred in eight (8/25, 32%) patients, including cannulation site bleeding in seven patients, and leg ischaemia requiring reperfusion cannula placement in one patient (see Supplementary Table 3). At discharge, 89% (16/18) of survivors recovered fully or had a mild disability (Cerebral Performance Category [CPC] scale 1) without additional complications. Two survivors with seizures were severely disabled (CPC scale 3) and were dependent on caregivers to assist with daily life activities.

### Subgroup Analysis According to the Timing of ECMO Treatment in Patients Who Experienced Cardiogenic Shock

**Figure 3:**
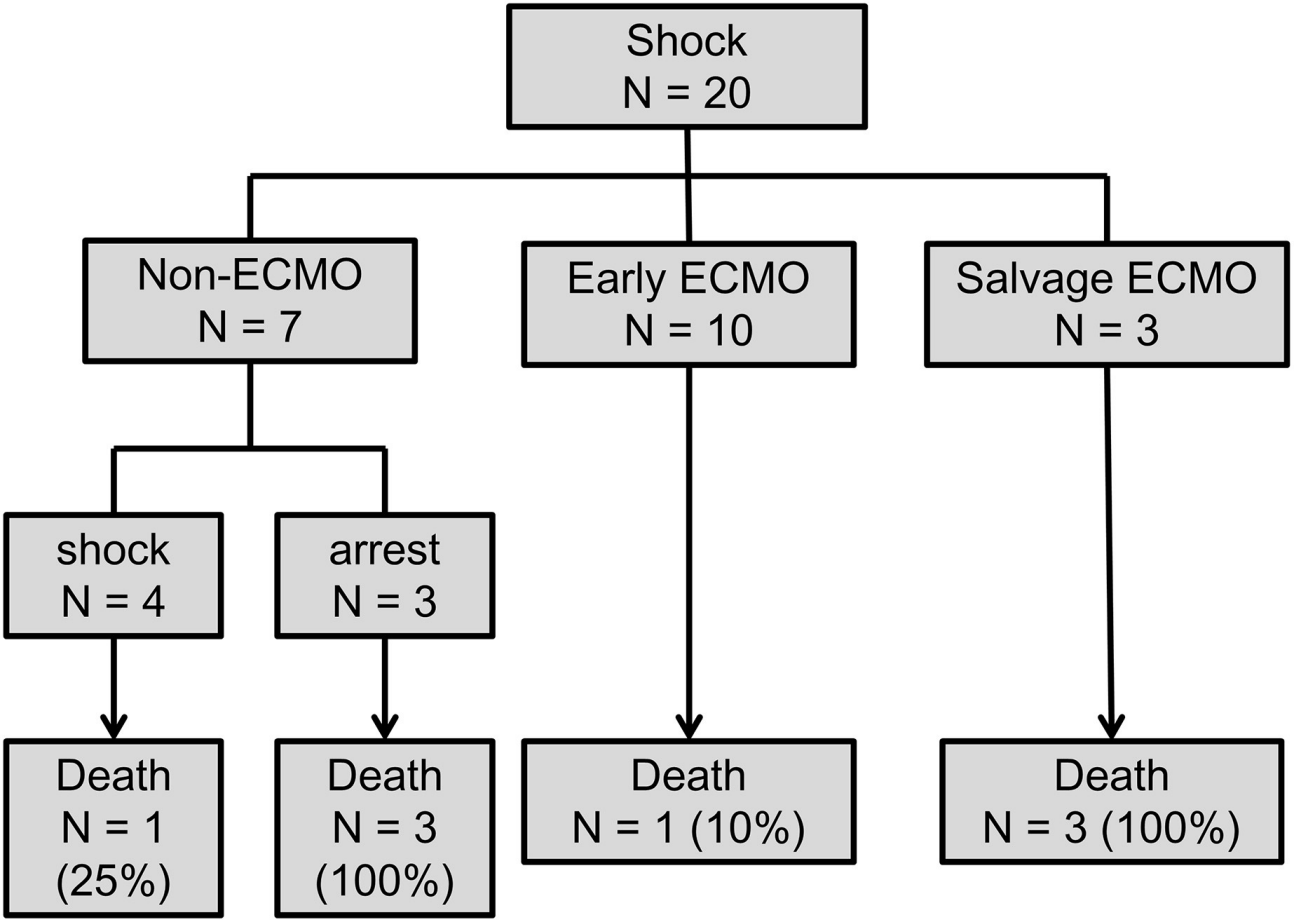
Flow chart of comparing the outcome in patients with and without earlier ECMO treatment. ECMO, veno-arterial extracorporeal membrane oxygenation.

**Figure 4:**
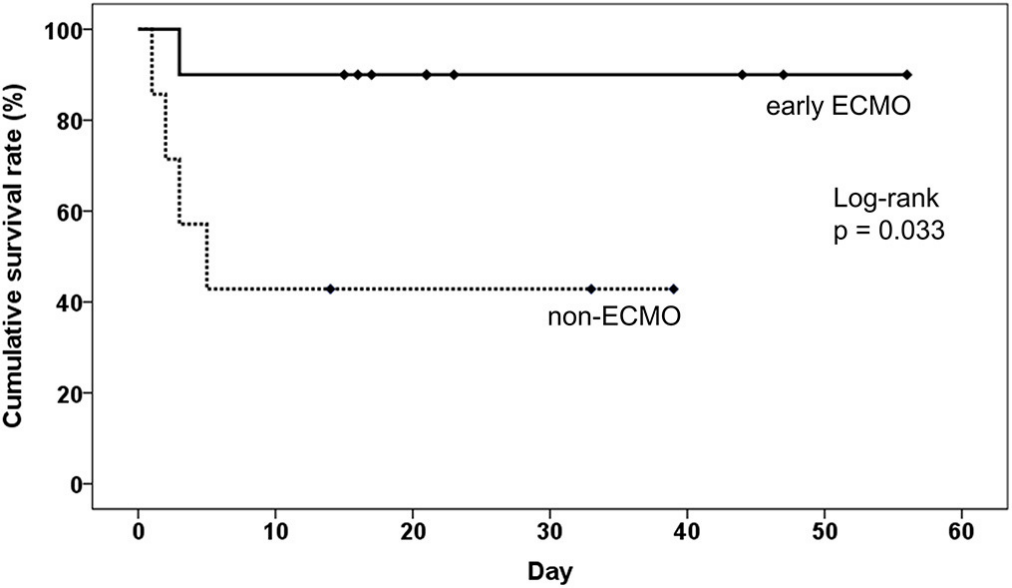
The impact of earlier ECMO treatment on the overall survival of pulmonary embolism patients without sudden cardiac arrest. ECMO, veno-arterial extracorporeal membrane oxygenation.

Subgroup analysis was designed to evaluate whether ECMO could be used as initial treatment in PE patients presenting with cardiogenic shock. In consideration of technical problems and real clinical practice for ECMO placement in patients who had an unexpected arrest within 30 min after a hypotensive episode, 20 (50%) patients with sudden cardiac arrest were excluded from the analysis. The enrolled patients were divided into three groups according to the timing of ECMO placement (Figure 3): non-ECMO group (*n* = 7); early ECMO group (*n* = 10), which was defined as ECMO initiation at the time of the cardiogenic shock episode; and salvage ECMO group (*n* = 3), which was defined as ECMO placement for prior cardiac arrest or during CPR. Compared with patients treated without ECMO, earlier ECMO treatment was associated with a reduced risk of cardiac arrest (*P* = 0.023). In-hospital mortality was also lower in the early ECMO group (10% vs. 57%, *P* = 0.036) (Table 4). Further, a log-rank test revealed a significantly higher cumulative overall survival in the early ECMO group (log-rank, *P* = 0.033) (Figure 4).

**Table 4:** Demographic characteristics, management and outcomes in subgroup (*n* = 17).

	**Non-ECMO** **(*n* = 7)**	**Early ECMO** **(*n* = 10)**	***P*-value**
**Characteristics**			
Age (years), mean (SD)	76.9 (5.3)	52.4 (18.6)	0.002
Male gender, *n* (%)	2 (28.6)	8 (80.0)	0.034
BMI (kg/m^2^), mean (SD)	27.5 (3.6)	26.4 (3.2)	0.534
Active cancer, *n* (%)	1 (14.3)	3 (30.0)	0.452
Major surgery, *n* (%)	1 (14.3)	9 (90.0)	0.002
**RV strain**			
RV strain on ECG, *n* (%)	4 (80.0) (*n* = 5)	7 (77.8) (*n* = 9)	0.923
RV dilation on echo, *n* (%)	1 (100.0) (*n* = 1)	9 (90.0)	0.740
RV/LV diameter ≥ 1.0 on CT, *n* (%)	2 (100.0) (*n* = 2)	8 (88.9) (*n* = 9)	0.621
Thrombolytic therapy, *n* (%)	2 (28.6)	4 (40.0)	0.627
**Outcomes**			
Progress to cardiac arrest	3 (42.9)	0 (0.0)	0.023
In-hospital mortality, *n* (%)	4 (57.1)	1 (10.0)	0.036

*BMI, body mass index; CPR, cardiopulmonary resuscitation; CT, computed tomography; ECG, electrocardiogram; ECMO, veno-arterial extracorporeal membrane oxygenation; IQR, interquartile range; RV, right ventricle; RV/LV, right-to-left ventricular; SD, standard deviation*.

## Discussion

Based on the Chang Gung Research Database, the largest multi-institutional electronic medical records for real-world epidemiological studies in Taiwan, we enrolled 421 patients with acute PE, of whom 40 were included in this study. The main findings of this study were as follows: (i) PE could rapidly progress to cardiac arrest before initiation of reperfusion therapy; (ii) cardiac arrest as the initial presentation was also common in patients with PE and resulted in poor outcomes, despite performing aggressive treatment; (iii) overall, ECMO treatment showed no survival benefit in unselected patients with high-risk PE; however, it had obvious effect on survival in those without cardiac arrest. Thus, the early use of ECMO in patients at the onset of shock followed by definitive treatment significantly reduces mortality from cardiac arrest and increase the overall survival rate.

Over the last two decades, the risk of mortality in patients with acute PE has decreased (12–14). This decrease may be attributed to growing physician awareness, better diagnostic tools, more frequent use of thrombolytics, and improved management of haemodynamically unstable patients. However, high-risk PE is still a life-threatening condition, with a mortality rate of 50% despite advances in diagnosis and treatment. According to the current 2019 European Society of Cardiology guidelines (2), systemic thrombolysis remains the standard reperfusion treatment for patients with haemodynamic instability. The class I recommendation is based on a systematic review of randomized trials performed before 2004 (15). Pooled data from five studies that included haemodynamically unstable PE patients suggested that thrombolysis was associated with a significant reduction in mortality in patients presenting with haemodynamic instability compared with anticoagulation (9.4% vs. 19.0%; OR 0.45).

Several problems have not been resolved in real-world clinical practice. First, the proportion of haemodynamically unstable patients treated with thrombolytic therapy is low. Based on two recent nationwide inpatient cohort studies in Germany (13) and the United States (16), only 15–30% of PE patients with haemodynamic instability receive systemic thrombolysis, which is similar to our result (30%). The most common reasons for patients not receiving thrombolysis were potential contraindications and comorbidities, such as older age, recent surgery, and active cancer (13). Second, even though thrombolytic agents can resolve thrombi within a few hours, it is still not a promising treatment for all patients with PE. Most deaths in patients with high-risk PE occurred within the first hour of symptom development, and the risk of death in patients treated with thrombolytic therapy was reported to be 15–30% (13, 16). Third, surgical embolectomy is an effective option to restore pulmonary artery perfusion and is recommended in patients with absolute contraindications to thrombolytic therapy or failed thrombolytic therapy (2). However, surgical embolectomy is rarely performed in clinical practice worldwide. Moreover, the overall hospital mortality rate following surgical embolectomy was 20–27%, which was higher than that in patients receiving systemic thrombolysis or catheter-directed therapy in national cohort studies (17, 18).

The mechanism of circulatory collapse and death in PE is acute RV failure. Reducing RV overload is key to reducing early death. ECMO is a rapid and reliable mechanical circulatory support device that decreases RV volume overload (5), and is also recommended as a treatment option for PE patients with refractory circulatory collapse or cardiac arrest. The overall survival rate of patients who receive ECMO for high PE has been reported to be 38–67% (5–8, 19). For patients who required CPR prior to ECMO initiation, the survival rate was only 13–27%, similar to our result (20%). According to the current guidelines, ECMO is suggested as a bridge to definitive reperfusion therapy. The class IIb recommendation of ECMO is based on several case series (6–8, 19, 20), and there are no case-control or cohort studies comparing ECMO to other treatments. In a systematic review of 50 articles encompassing 128 PE patients with ECMO support, 67.2% (86/128) of patients presented with cardiac arrest, and in-hospital mortality rates were 22% (20/91) (21). The excellent outcomes may be a result of the publication bias toward positive outcomes. Another systematic review included 16 uncontrolled case series demonstrated that in-hospital survival rate was 50–95%, with a major degree of heterogeneity (I2 > 70%) (22). Despite the lack of solid evidence, ECMO use has increased over time and has been shown to improve outcomes in high-risk PE based on national studies (23).

Nevertheless, ECMO implantation is an invasive procedure with a risk of adverse events, such as vascular injury during cannulation, limb ischemia, thromboembolic events, bleeding, infection, and rare life-threatening complications, such as great vessel perforation. The most common complication associated with percutaneous ECMO cannulation is limb ischemia, followed by cannulation site bleeding requiring transfusion or repair (24). In a study of 33 patients with high-risk PE treated with ECMO, 13 (40%) had cannulation site bleeding, and 2 (6%) had limb ischaemia (8). In our study, six (25%) patients had cannulation site bleeding requiring transfusion, one (4%) formed a pseudoaneurysm after cannula removal, and one (4%) had limb ischaemia requiring reperfusion catheter placement, which was lower than that in previous reports. ECMO treatment in higher-volume hospitals (> 100 cases per year) has been reported to have significantly lower mortality and complication rates (25).

To the best of our knowledge, this is the first cohort study to investigate the influence of ECMO on the survival of patients with high-risk PE. In this study, ECMO treatment showed no survival benefit in PE patients with cardiac arrest, but it showed excellent results in PE patients without cardiac arrest. There is no clinical value in the prediction of cardiac arrest in patients with PE at the onset of shock. Thus, we suggest that ECMO should be initiated as early as possible in high-risk PE patients. However, considering the complications of ECMO, a well-coordinated ECMO team may improve clinical outcomes and reduce ECMO-related complications (26).

This study had several limitations. First, this was a retrospective study, which may have led to a selection bias. ECMO was frequently performed in younger patients who could tolerate major surgery. This group had better functional performance and cardiopulmonary status, which may affect clinical outcomes. Second, because of the limited sample size, statistically significant differences were only hypothesis-generating. We could not exclude type 2 errors, particularly for categorical assessments. Third, our patients were treated in a high-volume ECMO tertiary referral teaching hospital, which may limit the generalisability of our observations. Fourth, only Asian patients were included, which may not represent all high-risk PE cases. Fifth, in our series, treatment with ECMO plus thrombolysis had a lower mortality rate compared with ECMO alone. There is a selection bias due to the fact that additional thrombolysis was frequently performed in patients without experiencing cardiac arrest or CPR-related severe brain injury. In addition, no patient underwent surgical embolectomy may also affect clinical outcomes. Although the data suggest that earlier ECMO treatment for patients without cardiac arrest may result in favorable outcomes, the definitive treatment to achieve best outcomes may not conclude in this study. Prospective, multicentre, large-scale studies could overcome this limitation.

In conclusion, in this retrospective cohort study, earlier ECMO treatment was associated with lower in-hospital mortality among haemodynamically unstable patients without cardiac arrest. However, patients with acute PE requiring CPR had an extremely high in-hospital mortality rate, and ECMO treatment showed no survival benefit. Our findings suggest that ECMO can be considered as an initial treatment option for patients with high-risk PE in higher-volume hospitals. Team based approach with well written protocols may improve outcomes for PE on ECMO better.

## Data Availability Statement

The original contributions presented in the study are included in the article/Supplementary Material, further inquiries can be directed to the corresponding author/s.

## Ethics Statement

The studies involving human participants were reviewed and approved by the Institutional Review Board of Chang Gung Medical Foundation (202000715B0). Written informed consent for participation was not required for this study in accordance with the national legislation and the institutional requirements.

## Author Contributions

Y-YC: conceptualization, formal analysis, writing—review and editing, and supervision. H-YT: methodology. K-RH: software. C-CW and K-RH: validation. H-YT and W-CL: investigation. W-CL, H-TY, and J-JS: resources. Y-TW and C-ML: data curation. H-YT and Y-TW: writing—original draft preparation. W-CL: visualization. Y-TW, H-TY, Y-CC, and J-JS: project administration. All authors contributed to the article and approved the submitted version.

## Conflict of Interest

The authors declare that the research was conducted in the absence of any commercial or financial relationships that could be construed as a potential conflict of interest.

## Publisher's Note

All claims expressed in this article are solely those of the authors and do not necessarily represent those of their affiliated organizations, or those of the publisher, the editors and the reviewers. Any product that may be evaluated in this article, or claim that may be made by its manufacturer, is not guaranteed or endorsed by the publisher.

## Footnotes

^1^ELSO Registry Data Definitions. Available online at: https://www.elso.org/Portals/0/Files/PDF/ELSOECLSRegistryForm8.0_2020.pdf

## Abbreviations

CI: confidence interval
CPC: cerebral performance category
CPR: cardiopulmonary resuscitation
CTPA: computed tomography pulmonary angiography
ECMO: veno-arterial extracorporeal membrane oxygenation
IQR: interquartile range
OR: odds ratio
PE: pulmonary embolism
RV: right ventricular
SD: standard deviation
V/Q: ventilation/perfusion.
