# Supplementary material for: Efficacy and Safety of Veno-Arterial Extracorporeal Membrane Oxygenation in the Treatment of High-Risk Pulmonary Embolism: A Retrospective Cohort Study
Source: Front Cardiovasc Med. 2022 Mar 2;9:799488. doi: 10.3389/fcvm.2022.799488 (PMC8924067; doi:10.3389/fcvm.2022.799488)
Supplement: Supplementary file 1 [file Table_1.docx]

**Table S1. Relative and absolute contraindications for thrombolysis**

|  | With thrombolysis  (n = 12) | Without thrombolysis  (n = 28) |
| --- | --- | --- |
| **Dismal outcome** |  |  |
| SCA with failed resuscitation* |  | 4 |
| **Absolute contraindications** |  |  |
| Prior intracranial hemorrhage |  | 4 |
| Malignant intracranial neoplasm |  | 1 |
| Recent intracranial/spinal surgery (within three weeks) |  | 6 |
| Severe brain injury after prolonged CPR | 3 | 11 |
| Ischemic stroke within three months |  | 1 |
| Active bleeding or bleeding diathesis |  | 1 |
| **Relative contraindications** |  |  |
| History of ischemic stroke more than three months |  | 2 |
| Prolonged (>10 minute) CPR | 3 | 1 |
| Major surgery less than three weeks | 5 | 3 |
| Recent (within two to four weeks) internal bleeding | 1 | 1 |
| Active peptic ulcer | 5 | 3 |
| No above contraindication | 1 | 1 |

CPR, cardiopulmonary resuscitation; SCA, sudden cardiac arrest.

* sudden cardiac arrest, defined as an unexpected arrest within 30 minutes after hypotensive episode
