# Supplementary material for: Efficacy and Safety of Veno-Arterial Extracorporeal Membrane Oxygenation in the Treatment of High-Risk Pulmonary Embolism: A Retrospective Cohort Study
Source: Front Cardiovasc Med. 2022 Mar 2;9:799488. doi: 10.3389/fcvm.2022.799488 (PMC8924067; doi:10.3389/fcvm.2022.799488)
Supplement: Supplementary file 2 [file Table_2.docx]

**Table S2. Summary of patients receiving thrombolysis (n = 12)**

| Case No. | Age, years  /Sex | Thrombolytic strategy (regimen) | Timing of thrombolysis | Cardiac arrest | ECMO indication | Outcome |
| --- | --- | --- | --- | --- | --- | --- |
| 1 | 81/F | CDT (rt-PA 100mg/6hr) | After CPR | Yes | - | Alive |
| 2 | 77/F | CDT (rt-PA 5mg bolus, 19mg/19h) | Shock | Yes | - | Dead |
| 3 | 81/F | ST (rt-PA 100mg/2h) | Shock | No | - | Dead |
| 4 | 42/M | ST (rt-PA 100mg/2h) | After ECMO | Yes | During CPR | Dead |
| 5 | 59/M | ST (urokinase infusion x 2 days) | After ECMO | Yes | During CPR | Alive |
| 6 | 83/M | CDT (urokinase infusion x 7 days) | After ECMO | No | Shock | Alive |
| 7 | 56/M | CDT (urokinase infusion x 4 days) | After ECMO | No | Shock | Alive |
| 8 | 66/F | CDT (rt-PA 62mg/4hr) | Shock | Yes | During CPR | Alive |
| 9 | 46/F | ST (rt-PA 10mg bolus) | After ECMO | Yes | Previous CPR | Dead |
| 10 | 72/M | CDT (urokinase infusion x 1 day) | After ECMO | No | Shock | Alive |
| 11 | 45/M | CDT (urokinase infusion x 2 days) | After ECMO | No | Shock | Alive |
| 12 | 35/F | CDT (urokinase infusion x 2 days) | After ECMO | Yes | During CPR | Alive |

CDT, catheter-directed thrombolysis; CPR, cardiopulmonary resuscitation; ECMO, veno-arterial extracorporeal membrane oxygenation; F, female; M, male; rt-PA, recombinant tissue-type plasminogen activator; ST, systemic thrombolysis.
