# Supplementary material for: Efficacy and Safety of Veno-Arterial Extracorporeal Membrane Oxygenation in the Treatment of High-Risk Pulmonary Embolism: A Retrospective Cohort Study
Source: Front Cardiovasc Med. 2022 Mar 2;9:799488. doi: 10.3389/fcvm.2022.799488 (PMC8924067; doi:10.3389/fcvm.2022.799488)
Supplement: Supplementary file 3 [file Table_3.docx]

**Table S3. Summary of patients receiving ECMO (n=25)**

| Case No. | Age, years | Sex | ECMO indication | ECMO insertion technique | ECMO duration, days | Major ECMO complications | Thrombolysis | Outcome |
| --- | --- | --- | --- | --- | --- | --- | --- | --- |
| 1 | 42 | M | during CPR | puncture | 20 | none | post-ECMO | Dead |
| 2 | 73 | M | shock | puncture | 12 | cannulation site bleeding | none | Alive |
| 3 | 59 | M | during CPR | open | 4 | cannulation site bleeding | post-ECMO | Alive |
| 4 | 83 | M | shock | puncture | 3 | none | post-ECMO | Alive |
| 5 | 70 | M | during CPR | puncture | 2 | none | none | Dead |
| 6 | 55 | M | during CPR | open | 6 | none | none | Dead |
| 7 | 56 | M | shock | puncture | 7 | none | post-ECMO | Alive |
| 8 | 30 | M | during CPR | open | 10 | cannulation site bleeding | none | Dead |
| 9 | 66 | F | during CPR | open | 9 | none | pre-ECMO | Alive |
| 10 | 59 | F | prior CPR | puncture | 4 | none | none | Dead |
| 11 | 80 | M | prior CPR | open | 3 | cannulation site bleeding | none | Dead |
| 12 | 26 | F | shock | puncture | 5 | none | none | Alive |
| 13 | 42 | M | shock | puncture | 2 | none | none | Dead |
| 14 | 46 | F | prior CPR | puncture | 1 | none | post-ECMO | Dead |
| 15 | 32 | F | shock | puncture | 2 | none | none | Alive |
| 16 | 43 | M | shock | puncture | 1 | cannulation site bleeding | none | Alive |
| 17 | 52 | M | shock | puncture | 7 | none | none | Alive |
| 18 | 51 | F | during CPR | open | 2 | cannulation site bleeding | none | Dead |
| 19 | 72 | M | shock | puncture | 6 | none | post-ECMO | Alive |
| 20 | 45 | M | shock | puncture | 3 | none | post-ECMO | Alive |
| 21 | 65 | M | during CPR | puncture | 3 | none | none | Dead |
| 22 | 45 | M | during CPR | open | 4 | leg ischemia | none | Dead |
| 23 | 62 | M | during CPR | open | 1 | none | none | Dead |
| 24 | 35 | F | during CPR | puncture | 9 | cannulation site bleeding | post-ECMO | Alive |
| 25 | 28 | F | during CPR | open | 2 | none | none | Dead |

CPR, cardiopulmonary resuscitation; ECMO, Veno-arterial extracorporeal membrane oxygenation; F, female; M, male.
